# Supplementary material for: Stimulus-dependent delay of perceptual filling-in by microsaccades
Source: J Vis. 2025 Jul 7;25(8):8. doi: 10.1167/jov.25.8.8 (PMC12248979; doi:10.1167/jov.25.8.8)
Supplement: Supplement 1 [file jovi-25-8-8_s001.pdf]

Stimulus-dependent delay of perceptual filling-in by microsaccades

Max Levinson *et al.*

Supplementary Materials

Figs. S1 to S3
Tables S1 to S3

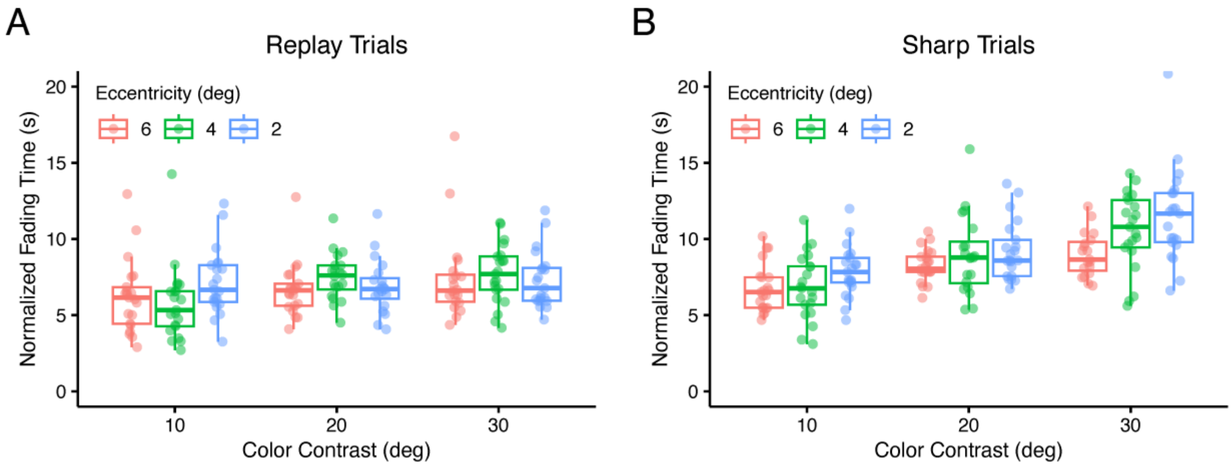

Fig. S1. Catch trial filling-in times. Normalized filling-in times for two categories of catch stimuli: **(A)** replay of the filling-in experience and **(B)** sharp, 1-pixel width boundaries between center and periphery. Boxplots show median, 25th and 75th percentiles.

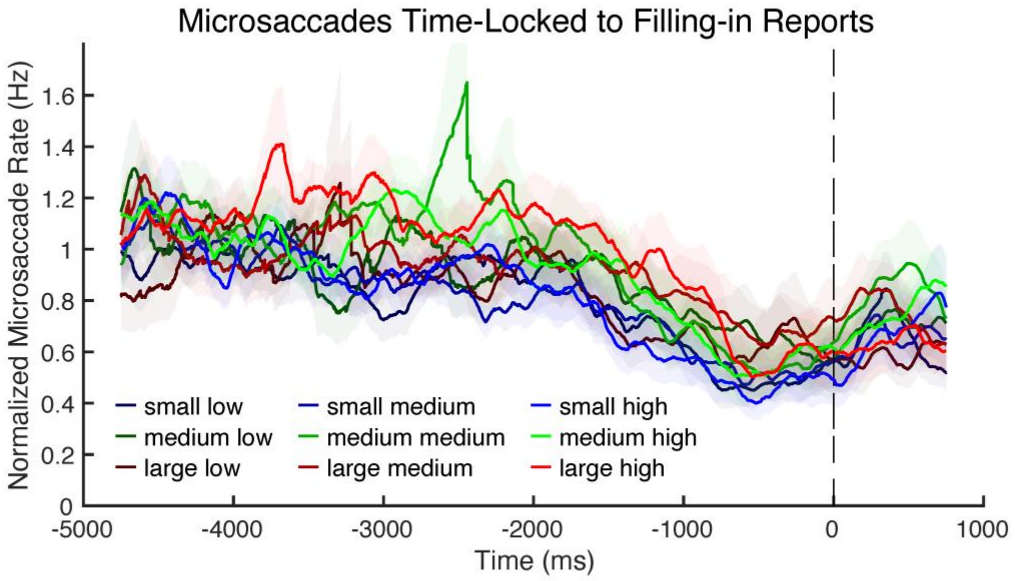

Fig. S2. Microsaccade rate variability across stimulus conditions. Data are aligned to the subjective report of perceptual filling-in (time 0). Curves represent different combinations of boundary size (small, medium, large) and color contrast (low, medium, high). Shaded areas indicate standard error of the mean.

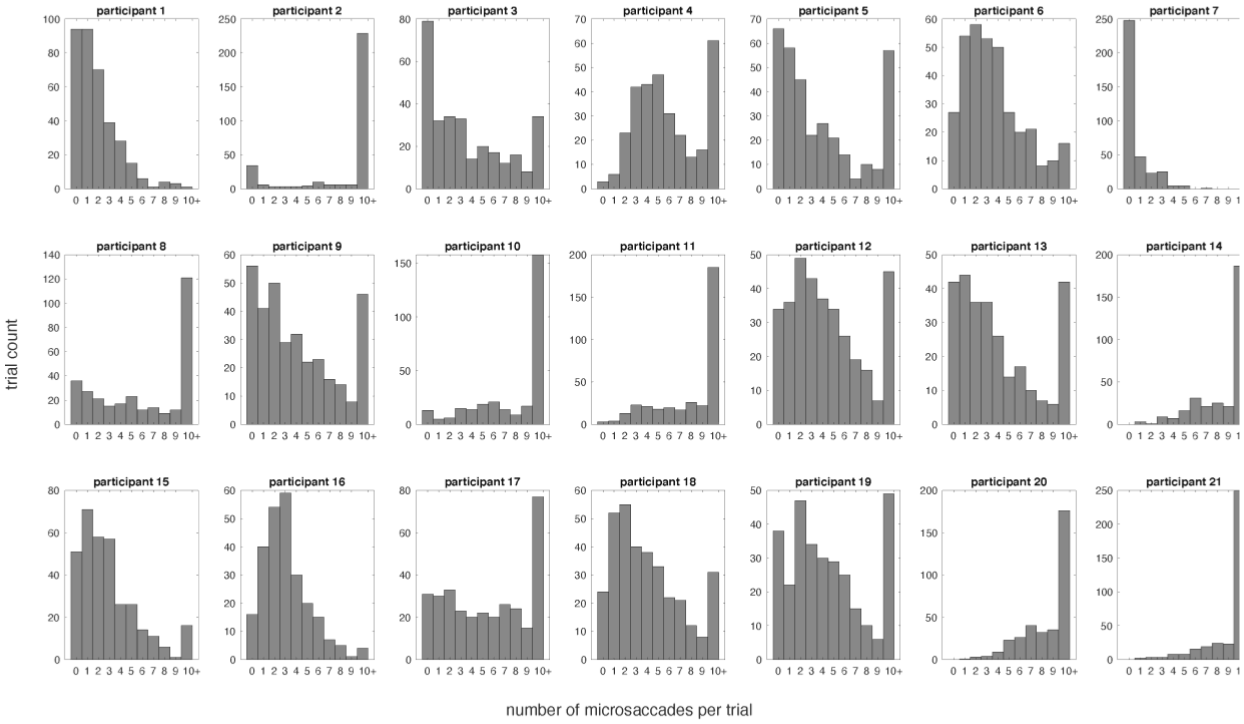

Fig. S3. Distributions of microsaccade counts within each trial, plotted for each participant separately.

Table S1. Fixed effects from a linear mixed-effect model of filling-in time by stimulus and trial parameters.

Parameter	Estimate	99.17% Percentile CI
intercept	0.566	0.441, 0.701
contrast	0.133	**0.0995, 0.166 ***
eccentricity	-0.0930	**-0.125, -0.0603 ***
trial_num	-0.107	**-0.128, -0.0859 ***
contrast : eccentricity	0.00831	-0.0319, 0.0526
contrast : trial_num	-0.0203	-0.0397, 0.000423
eccentricity : trial_num	-0.0116	-0.0311, 0.00999

*: the percentile bootstrap confidence interval (CI), Bonferroni-corrected across fixed effect parameters, does not contain 0 ($\alpha = 0.0083$). The intercept is presented for reference but not evaluated for statistical significance.

Table S2. Fixed effects from a linear mixed-effect model of filling-in time by stimulus, trial, and eye movement parameters.

Parameter	Estimate	99.29% Percentile CI
intercept	0.274	0.0994, 0.427
contrast	0.111	**0.0810, 0.141**
eccentricity	-0.0747	**-0.0998, -0.0503**
trial_num	-0.105	**-0.122, -0.0865**
ms_presence	0.307	**0.181, 0.466**
num_blinks	0.274	**0.210, 0.349**
ocular_drift	0.0293	-0.0222, 0.0803
contrast : trial_num	-0.0154	-0.0313, 0.000384

This model includes the significant parameters identified in Table S1, plus main effects of eye movements.

*: the percentile bootstrap confidence interval (CI), Bonferroni-corrected across fixed effect parameters, does not contain 0 (α = 0.0071). The intercept is presented for reference but not evaluated for statistical significance.

Table S3. Summary of microsaccade rates for each participant.

Participant Number	Microsaccade Rate (Hz)	# Trials With 0 Microsaccades
1	0.26	93
2	2.01	33
3	0.49	78
4	0.81	3
5	0.60	36
6	0.73	26
7	0.15	246
8	1.16	25
9	0.54	49
10	0.97	13
11	1.86	2
12	0.98	22
13	0.56	7
14	1.25	0
15	0.38	43
16	0.92	16
17	0.61	28
18	0.62	13
19	0.54	38
20	1.35	0
21	1.90	0
